# Non-Animal Hyaluronic Acid from *Tremella fuciformis*: A New Source with a Structure and Chemical Profile Comparable to Hyaluronic Acid

**DOI:** 10.3390/foods14081362

**Published:** 2025-04-15

**Authors:** Rebecca Galla, Simone Mulè, Sara Ferrari, Francesca Parini, Annalisa Givonetti, Maria Cavaletto, Ivana Miletto, Geo Paul, Giovanni Battista Giovenzana, Leonardo Marchese, Claudio Molinari, Francesca Uberti

**Affiliations:** 1 Noivita S.r.l.s., Spin Off of University of Piemonte Orientale, Via Solaroli 17, 28100 Novara, Italy; 2 Department for Sustainable Development and Ecological Transition, University of Piemonte Orientale, Piazza Sant’Eusebio 5, 13100 Vercelli, Italy; 3 Department of Pharmaceutical Sciences, University of Piemonte Orientale, Largo Guido Donegani 2/3, 28100 Novara, Italy; 4 Department of Science and Technological Innovation, University of Piemonte Orientale, Viale Teresa Michel 11, 15100 Alessandria, Italy

**Keywords:** *Tremella fuciformis* extract, non-animal hyaluronic acid, chemical composition, physical composition, polysaccharide composition

## Abstract

*Tremella fuciformis* is high in polysaccharides, which have a structure made up of a straight chain of (1→3) α-D-mannan and side chains of glucuronic acid, xylose, and fucose. This study aimed to evaluate whether the non-animal hyaluronic acid extracted from *Tremella fuciformis* can maintain the chemical and physical characteristics of hyaluronic acid that ensure its biological functionality. Chemical and physical analyses such as hyaluronic content, screening of metals, purity, pH, nuclear magnetic resonance (NMR), Fourier transform infrared spectroscopy (ATR/FTIR), and MALDI-TOF were performed. Chemical characterisation revealed that the most abundant polysaccharide in the extract was hyaluronic acid, accounting for ca. 87.76%, with a molecular weight above 2000 kDa. In addition, ATR/FTIR and NMR spectroscopy and MALDI-TOF analysis confirmed that *Tremella fuciformis* extract is a source of non-animal hyaluronic acid. In summary, every molecular attribute examined played a significant role in determining the functional qualities of the extract, indicating that a thoughtful choice of extraction technique can enhance its advantages.

## 1. Introduction

The cultivation of edible mushrooms has a long history in Asia. They are important to diets worldwide as they are rich in nutrients and medicinal benefits [1]. In addition, they possess secondary metabolites such as terpenes and proteins with antimicrobial, antiviral, antitumor, antithrombotic, and immune properties [2]. Mushroom-derived polysaccharides, particularly β-glucans, are gaining interest among researchers and food manufacturers for their antioxidant, antidiabetic, anticarcinogenic, and immunomodulatory effects and multiple health benefits [3].

*Tremella fuciformis* Berk, the fruiting body of the basidiomycete fungus *Tremella*, has been used for thousands of years in traditional Chinese medicine for its remarkable therapeutic properties in skin care, immune system strengthening and disease prevention. The polysaccharide of *Tremella fuciformis* (TFPS), identified as the main bioactive component of *Tremella fuciformis* present in the fruiting body, spores, mycelia, and fermentation liquor, shows multiple physiological and health-promoting effects, such as immune function enhancement, antitumor, antioxidation, anti-ageing, hypoglycaemia, hypolipidemia, neuroprotection, and other effects [4]. *Tremella fuciformis* is naturally rich in polysaccharides, which may contribute to anti-ageing and antioxidant effects due to its ability to prevent cellular damage caused by free radicals [5]. The chemical structure of polysaccharides consists of a linear backbone of (1→3) α-D-mannan with side chains composed of glucuronic acid, xylose and fucose. The estimated ratio of mannose, fucose, xylose, and glucuronic acid in *Tremella fuciformis* is 9:1:4:3, with glucuronic acid accounting for 17.6% of the polysaccharide content [6,7]. Moreover, the acid polysaccharide derived from the fruiting body of *Tremella fuciformis* through hydrothermal treatment is identified as a high molecular weight polysaccharide exceeding 10^6^ Da, primarily comprising mannose, glucuronic acid, glucose, xylose, and fucose (with a mass ratio of monosaccharides at approximately 6.8:1:0.15:1.5:0.6). Especially, different experimental conditions can result in various fractions of TFPS, so that TFPS is a mixture of different polysaccharides with molecular weights between 5.82 × 10^5^ Da and 3.74 × 10^6^ Da. The characterised chemical structure of TFPS consists of a linear backbone (1→3) of α-D-mannose with highly branched side chains of β-D-xylose, α-D-fucose and β-D-glucuronic acid [8]. Structural modification of polysaccharides primarily involves exploiting their inherent reactive groups, such as the hydroxyl, carboxyl, and amine groups, to introduce new functional groups through chemical methods. The process of chemically modifying polysaccharides encompasses various techniques, including acetylation, carboxymethylation, sulfation, phosphorylation, alkylation, and selenisation [9]. The most common chemical modification of TFPS is sulfation, which has improved its biological activities and water solubility [10].

The conservation of the fresh fruiting body of *Tremella fuciformis* poses challenges due to its susceptibility to browning, changes in olfactory characteristics, and deterioration, which may transpire even under refrigeration. Consequently, the assurance of bioactive compounds’ stability and aroma regulation are critical concerns within the industry. The investigation undertaken by Lin et al. [11] analysed the proportions and variations in the composition of *Tremella fuciformis* polysaccharides produced via an array of desiccation methodologies, including freeze-drying, cold air drying at 18 °C, and hot air drying at 50 °C. Furthermore, their study explored the kinetic parameters of the endothermic reactions pertinent to *Tremella fuciformis* polysaccharides employing diverse thermal analytical techniques, such as differential scanning calorimetry [12] and thermogravimetry [13], which enabled discernment of the characteristics of additives suitable for incorporation in cosmetics, health food products, biomaterials, and various other applications. Also, the growth of *Tremella fuciformis* is subject to the influence of multiple elements (such as temperature, light, and water), leading to potential fluctuations in the quality of polysaccharides. Interestingly, employing the submerged fermentation technique effectively regulates these delicate factors [14]; thus, manipulating fermentation parameters can impact the polysaccharide output of *Tremella fuciformis* [15]. In general, the isolation of polysaccharides from marine and terrestrial sources includes enzymatic hydrolysis [16]. Still, in the case of *Tremella fuciformis*, polysaccharides are generally extracted by subjecting pulverised fruiting bodies to a prolonged period of stirring in hot water [17,18]. The method of polysaccharide extraction from *Tremella fuciformis*, as outlined in the extant literature, involves initial immersion of the dried mushroom in water, followed by a series of subsequent mixing, heating, cooling, and centrifugation steps [19]. Nonetheless, the extraction process has the capacity to exert an influence on such factors as the content, quality and chemical and physical composition of the polysaccharides [20].

The prevailing methodology for isolating TFPS is characterised by ethanol precipitation; however, this method is associated with extended extraction times and considerable depletion of polysaccharides. Additionally, the polysaccharides obtained are often tainted with various contaminants, such as pigments, proteins, and nucleic acids, which necessitates further purification procedures [21]. The fundamental objective of TFPS extraction is to optimise extraction efficiency while preserving the integrity of the polysaccharide’s active structure. Consequently, the selection of an appropriate extraction technique is of paramount importance. Various extraction methodologies significantly influence polysaccharides’ efficacy, physicochemical characteristics, and biological activities [22]. The scientific literature indicates that the extraction rate of TFPS can be increased 3–4 times by using two or more extraction methods compared to a single extraction method. For example, repeated freezing and thawing steps combined with the double water phase method are used to extract the TFPS from the fermentation broth, which enabled the removal of most proteins and pigments and increased the extraction rate of the polysaccharide to over 80% [21,23]. After choosing the extraction method, the supernatant is collected by centrifugation or filtration, and the residue is extracted, usually three times. Notably, despite ethanol limitations, the extract is then concentrated to a quarter of its original volume [24] and precipitated using three volumes of absolute ethanol at 4 °C for 24 h. The precipitate is collected by centrifugation and freeze-drying [25] or low-temperature drying [18] to obtain the crude polysaccharide. In the present study, diluting the ethanol as much as possible was preferred to obtain a minimum concentration sufficient to precipitate the extract without altering its structure and content. Furthermore, the ethanol was systematically diluted until its complete removal, signifying an advancement in the extraction process beyond the extant literature [15,26,27].

In summary, a purification process is required at the end of the extraction process to get an extract containing only polysaccharides. The purification process of the unrefined TFPS commonly utilises ion-exchange chromatography to segregate neutral and acidic polysaccharides, with elution performed using water and gradients containing different sodium chloride concentrations. The underlying concept is linked to the equilibrium between ion exchange and molecular weight impacts. Additionally, gel filtration chromatography can segregate polysaccharides based on their molecular weights. Polysaccharides with higher molecular weights facilitate a quicker separation from chromatography columns [28]. Purified polysaccharides can be analysed using high-performance liquid chromatography (HPLC) to determine their purity and molecular weight.

As reported in the literature, they can be analysed by ultraviolet spectroscopy (UV), infrared spectroscopy (IR), gas chromatography (GC), mass spectrometry (MS), and nuclear magnetic resonance (NMR) to determine the structure of polysaccharides [29].

In the present study, the application of a sequential hydrolysis extraction method and ethanol precipitation from a mushroom represents an innovation. Specifically, the innovation is to be able to obtain HA from grinding a mushroom as is without chemical additions, resulting in a high molecular weight HA contrary to what has always been obtained (low molecular weight HA). Therefore, based on these premises, the purpose of the following study was to characterise the hyaluronic acid (HA) extract derived from *Tremella fuciformis* through chemical and physical analyses to confirm the presence and purity of hyaluronic acid and determine its structural properties. This study was undertaken to complement previous observations at the joint level [30] and enhance the extract’s chemical and physical valence by supporting its biological effects.

## 2. Materials and Methods

### 2.1. The Materials and Chemical Reagents

**Figure 1:**
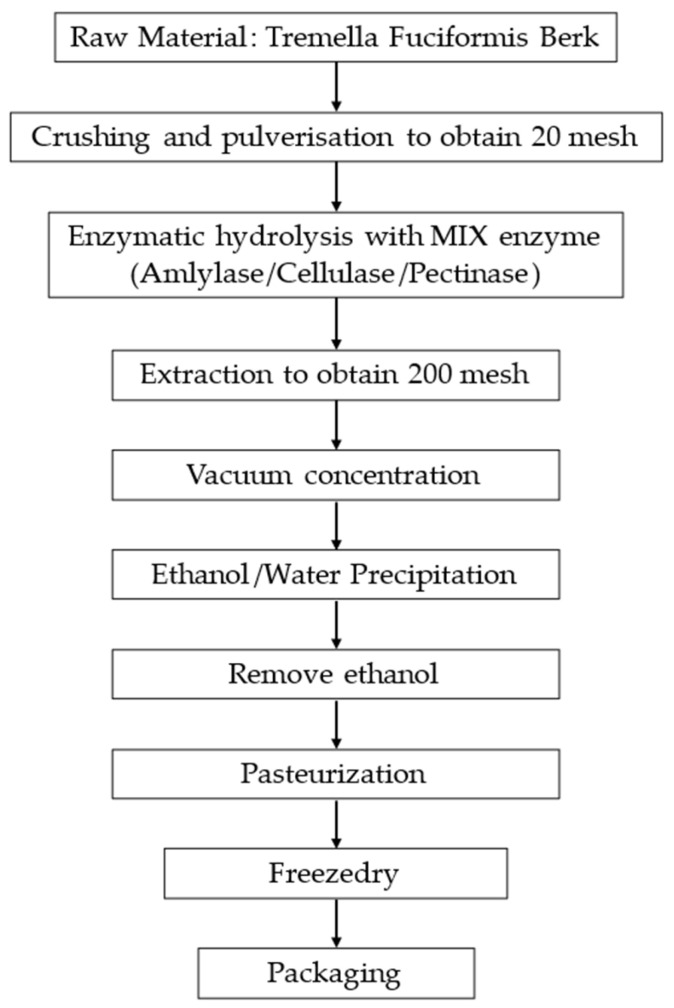
Flowchart of key points of the non-animal hyaluronic acid extraction process [31].

Non-animal hyaluronic acid was obtained from White Tremella (Silver Ear), a traditional foodstuff with medicinal applications in China [4]. The production process involves several steps necessary to get a final extract (named GreenIuronic^®^) and includes a new technology based on patents N° WO/2020/245809 and N° WO2021/250566A1 from Vivatis Pharma GBHE, Grüner Deich 1–3, 20,097 Hamburg, Germany. The process involves extraction, purification, and refining with an alcohol solution, sieving, and crushing (Figure 1). The resulting powder is packed, tested for metals, and stored [31,32]. All chemicals and reagents used in this study were purchased from Merck Life Science (Rome, Italy), MilliporeSigma (Burlington, MA, USA), and Sigma-Aldrich (Darmstadt, Germany). In addition, all chemicals and reagents were of analytical grade.

### 2.2. Non-Animal Hyaluronic Acid Extraction from Tremella fuciformis

The methodology employed for extracting non-animal hyaluronic acid from *Tremella fuciformis* constitutes a multi-faceted process; the extraction procedure, under patent, is delineated into distinct stages [31]. The preliminary phase involves identifying *Tremella fuciformis* fungi deemed suitable for maturation and growth, which will be utilised in the extraction endeavour. Following this initial phase, the selected fungal biomass underwent a fragmentation and pulverisation step, obtaining a 20 mesh powder, and then underwent an enzymatic hydrolysis step conducted at 95 °C for 3–10 h using an aqueous solvent infused with proteolytic enzymes such as amylase, pectinase, and cellulase. Resulting in a 200 mesh product, which was then concentrated by creating a vacuum. At the end of the extraction and concentration phases, a precipitation phase was carried out for 4 h using food-grade ethanol diluted to 80% in a ratio of 3:4; once the precipitate was formed, the precipitating agent was removed by heating to below room temperature (about 15 °C) and diluting with water. Finally, the extract was concentrated and dried to obtain non-animal hyaluronic acid. A schematic representation of the extraction process is shown in Figure 1.

### 2.3. Total Polysaccharides Content Colorimetric Assay

6 mg of raw sample (*Tremella fuciformis*) was dissolved in 1 mL of deionised water (carbohydrate solution) and mixed with 1 mL of 5% (*w*/*w*) aqueous phenol solution (phenol, Merck Life Science, Rome, Italy) prepared immediately before the use in a test tube. Subsequently, 5 mL of concentrated sulfuric acid (99%, Merck Life Science, Rome, Italy) was added rapidly to the mixture. After allowing the test tubes to stand for 10 min, they were vortexed for 30 s and placed for 20 min in a water bath at room temperature for colour development. Then, light absorption at 490 nm was recorded on a spectrophotometer (Infinite 200 Pro MPlex, Tecan, Männedorf, Switzerland). Reference solutions (6 mg/mL of saccharose, Merck Life Science, Rome, Italy) were prepared identically to the above, except that the 2 mL aliquot of carbohydrate was replaced by bidistilled water. The phenol (Merck Life Science, Rome, Italy) used in this procedure was redistilled, and 5% phenol in water (*w*/*w*) was prepared immediately before the measurements. The calibrations were performed using standard solutions prepared from the corresponding high-purity sugar saccharose. The UV was programmed to produce a calibration curve with the following concentrations: 10, 20, 30, 40, 50, 60, and 70 mg/L. The solution was measured in triplicate and expressed as mean (%*w*/*w*) ± SD [33].

### 2.4. Hyaluronic Acid (HA) Content Colorimetric Determination

The samples’ hyaluronic acid (HA) content was determined as reported in the literature [30,34] with a few modifications. 6 mg of raw material (*Tremella fuciformis*) was dissolved in 1 mL of deionised water, and 10 µL of resuspended samples were displaced in new Eppendorf tubes and diluted with 190 µL deionised water. Then, 1.2 mL of sulfuric acid (Merck Life Science, Rome, Italy) and 0.0125 M tetraborate (Merck Life Science, Rome, Italy) were displaced in Eppendorf tubes containing diluted samples, shaken for 20 s, and then boiled at 100 °C for 5 min. After cooling on ice, 20 µL of 0.15% hydroxydiphenyl (Merck Life Science, Rome, Italy) (dissolved in 0.5% NaOH, Merck Life Science, Rome, Italy) was added and stirred; successively, 100 µL of each sample was placed in a 96 multi-well plate, and absorbance at 340 nm was measured using a spectrophotometer (Infinite 200 Pro MPlex, Tecan, Männedorf, Switzerland). The data were compared to a calibration curve generated using glucuronic acid (standard curve range from 0 to 6000 µg/mL, Merck Life Science, Rome, Italy). The results were expressed as mean (%*w*/*w*) ± SD.

### 2.5. Water Content Determination

The analysis was performed following the Karl Fischer method [35]. The percentage of water concentration in the sample was determined by KF titration using a Mettler Toledo model C20 coulometric KF titrator and Aquastar CombiCoulomat fritless methanol solution (MilliporeSigma, Burlington, MA, USA) as the reagent. The KF titrator was qualified for use before analysis using Hydranal^®^ Water Standard 10.0 (Honeywell Fluka, Mexico City, Mexico).

### 2.6. N-Acetyl-Glucosamine Content Determination

The HPLC system (Agilent, Santa Clara, CA, USA) separated and detected Acetylgalactosamine (GalNAc) and N-acetylglucosamine (NAG). A C18 Luna column (Phenomenex, Castel Maggiore, BO, Italy) was used to separate GalNAc and NAG at 30 °C in a column oven. The mobile phase consisted of water and methanol (35:65, *v*/*v*) and was pumped at a 0.3 mL/min flow rate. The isocratic mode was applied. The injection volume was 20 µL. The reading wavelength was 195 nm. GlcNAc and NAG working standard solutions were prepared daily from 25 to 200 µg/mL and 10 to 160 µg/L, respectively, by diluting in the mobile phase [36].

### 2.7. Screening of Metals and Sodium Content

Metals and sodium were analysed using the United States Pharmacopeia (USP) method 233 [37]. The procedure for determining metal concentrations aims to detect elemental impurities, which are generally detectable by inductively coupled plasma mass spectrometry (ICP-MS). A 100 mg sample was weighed into a 50 mL tube, dissolved in 5 mL ultrapure water, and sonicated for 15 min. After sonication, the volume was adjusted to 50 mL by adding ultrapure water. Thus, the solution was analysed using iCAP™ PRO XP ICP-OES spectrometer (Thermo Fisher Scientific Inc., Waltham, MA, USA).

### 2.8. Experimental Protocol to Determine Molecular Weight

The molecular weight of non-animal hyaluronic acid was determined using 1% agarose gel, following the method reported in the literature for HA determination [30,38], with a few modifications. Briefly, 0.3 g agarose (Merck Life Science, Rome, Italy) was dissolved in 30 mL of TAE buffer (48.5 g tris base, 11.4 mL acetic acid, and 0.5 M ethylenediaminetetraacetic acid, EDTA, pH 8; all substances were purchased from Merck Life Science, Rome, Italy). The solution was heated for 30 s in a microwave at high power. The gel was poured into the holder and solidified before performing a pre-run at 100 V for 45 min, using the Mini-Sub Cell GT System (Bio-Rad, Hercules, CA, USA). In the meantime, samples were prepared by dissolving 3 mg of raw samples in 1 mL of TAE buffer 1×. Before running the gel, 16 µL of the resuspended sample was put in a new Eppendorf tube, and 4 µL of loading buffer (0.2% Bromophenol Blue, 1 mL of TAE 1×, and 8.5 mL of glycerol, which were purchased from Merck Life Science, Rome, Italy) was added to each sample and the molecular weights (10 µL of Select-HA HiLadder, Echelon Biosciences, Tebu-Bio Srl, Magenta, Italy). The samples were run at 100 V until they reached 1 cm from the end of the gel. Then, the gel was hydrated in ultrapure water for 24 h at room temperature in agitation, and then the gel was placed in 30% ethanol with 0.015% Stains All dye (Merck Life Science, Rome, Italy) for 24 h in the dark. The gel was decoloured for 30 min in ultrapure water in the dark before image acquisition using ChemiDoc™ Touch Imaging System (Bio-Rad, Hercules, CA, USA). The image obtained was analysed by Image Lab 3.0 software (Bio-Rad Hercules, CA, USA).

### 2.9. Particle Size Distribution

The test product was solubilised in an organic solvent and analysed by HPLC (Agilent, Santa Clara, CA, USA) with a GPC detector (Agilent 1260, Agilent, Santa Clara, CA, USA). The molecular weight assessment was performed by analysing reference materials at different molecular weights, in particular 194 Da, 1010 Da, 8500 Da, 19,330 Da, 141,700 Da, 427,500 Da, 1,044,000 Da, 1,250,000 Da, and 2,500,000 Da.

### 2.10. pH Determination USP 791

Following the standard protocol, one gram of the sample was weighed, dissolved in 100 mL of ultrapure water, and continuously agitated at room temperature. Once the material was fully dissolved, a pH assay was performed while the sample was continuously stirred [39]. The pH50 VioLab pH meter with XS 201T DHS digital electrode and integrated temperature sensor was used for detection (range: pH: 0.00–14.00; mV: 1000; temperature: 0–100.0 °C).

### 2.11. Purity

The purity of non-animal hyaluronic acid was determined by HPLC (HPLC instrument from Shimadzu, Kyoto, Japan) analysis using the method reported in the literature for HA detection [30]. Briefly, 20 μL of TRIS buffer (3.0 g TRIZMA base, 4.0 g sodium acetate trihydrate, 1.46 g sodium chloride, and 50 mg crystalline bovine serum albumin dissolved in 100 mL of 0.12 M HCl, pH 7.3 with 6 M HCl; all substances were purchased from Merck Life Science, Rome, Italy), 30 μL of chondroitinase AC solution (diluted to 10 U/mL with water), and 20 μL of the sample solution test (200 mg of raw material dissolved in 100 mL of ultrapure water) were pipetted into a conical 1.5 mL vial. The vial was placed in a warm water bath at 37 °C for 3 h. After cooling at room temperature, the sample was diluted to 1 mL by adding 930 μL of mobile phase A, and the mixture was analysed by HPLC-UV and HPLC-HRMS systems. A control solution was prepared by replacing the enzyme aliquot with TRIS buffer.

Specifically, for the HPLC-UV method, a column from Phenomenex Synergi Polar 4 µm 150 × 4.6 mm was preceded by a Security Guard Polar and kept at room temperature. The mobile phase A was composed of 340 mg of tetrabutylammonium bisulfate dissolved in 1000 mL of water HPLC grade, while the mobile phase B was composed of 340 mg of tetrabutylammonium bisulfate dissolved in 330 mL of water HPLC grade, then after the solution is at room temperature, brought to 1000 mL with acetonitrile. For the reading, a volume of injection of 30 µL was used, a flow rate of 1.1 mL/min, and a wavelength of 240 nm. The gradient elution program used is reported in Table A1 (Appendix A).

On the other hand, for the HPLC-HRMS method, a column from Phenomenex Synergi Polar 4 µm 150 × 2.0 mm was preceded by a Security Guard Polar and kept at room temperature. The mobile phase A comprised 0.1% formic acid in ultrapure water, while the mobile phase B comprised 0.1% formic acid in acetonitrile. For the reading, a volume of injection of 5 µL was used and a flow rate of 0.200 mL/min. The gradient elution program implemented is reported in Appendix A (Table A2).

### 2.12. NMR Analysis

A solid-state ^13^C magic angle spinning NMR spectrum was acquired on a Bruker Avance III 500 spectrometer (Fällanden, Switzerland) equipped with a wide bore 11.75 T magnet, operating at frequencies of 500.13 MHz for ^1^H and 125.77 MHz for ^13^C. A 4 mm triple-resonance probe with magic angle spinning (MAS) was used in all experiments. The samples were packed on a Zirconia rotor and spun at an MAS rate of 11–15 kHz. For the ^13^C cross-polarization (CP) MAS experiments, 55 and 28 kHz RF fields were used for initial proton excitation and decoupling, respectively. During the CP period, the ^1^H RF field was ramped using 100 increments, whereas the ^13^C RF fields were maintained at a constant level. The protons were decoupled from the carbons during the acquisition using a two-pulse phase-modulated decoupling method. A moderate ramped RF field of 62 kHz was used for spin locking, while the carbon RF field was matched to obtain the optimal signal [40]. The relaxation delay between accumulations was 5 s, and the CP contact time was 0.5 ms. All chemical shifts are reported using a δ scale and are externally referenced to tetramethylsilane at 0 ppm.

### 2.13. Attenuated Total Reflection (ATR) Fourier Transform Infrared Spectroscopy (FTIR) Analysis

The analysis was performed using a Bruker Alpha II instrument (Bruker Optics, Rosenheim, Germany) equipped with an ATR accessory with a monolithic diamond crystal and a DTGS detector. The instrument operated in the 4000–450 cm^−1^ range at a resolution of 4 cm^−1^. OPUS Software (Release 8.7) was used to process the ATR-FTIR spectra.

### 2.14. Polysaccharides Spectrum by Matrix-Assisted Laser Desorption/Ionisation Time-of-Flight (MALDI-TOF)

To verify a correct enzymatic extraction process, samples from different stages were analysed; in particular, 3.0 µL of raw material (10 mg of raw material dissolved in 1 mL of ultrapure water) were mixed with 3.0 µL of matrix solution (20 mg/mL 2,5-Dihydroxybenzoic acid dissolved in 30:70 (*v*/*v*) acetonitrile: 0.1% TFA in water, 1 mM NaCl; all substances were purchased from Merck Life Science, Rome, Italy). 0.5 µL of each mix was spotted on a ground steel MTP 384 target plate (Bruker Daltonics GmbH, Bremen, Germany) in triplicate and let to dry at room temperature overnight. MALDI-TOF spectra were acquired by an Ultraflextreme mass spectrometer (Bruker Daltonics GmbH, Bremen, Germany) equipped with a Smart-beam 2 Nd: YAG laser operating at 355 nm, using FlexControl software (version 3.3, Bruker Daltonics GmbH, Bremen, Germany). Samples were analysed linearly, and positive ion acquisition was achieved in a mass-to-charge ratio of 300–5000 *m/z*. The mass spectra obtained are 6000 shots for each sample; the random walk feature with partial sample mode was activated. This analysis was performed according to the data reported in the literature [41,42].

### 2.15. Statistical Analysis

The experimental procedures were conducted with three independent analyses, with the resulting data presented as mean values accompanied by their respective standard deviations. The statistical significance of the multiple averages was assessed using the variance analysis methodology using the statistical software Prism GraphPad 9.5.0. The statistical significance threshold was set at *p* < 0.05.

## 3. Results

### 3.1. Chemical Characterisation

#### 3.1.1. Analyses of Non-Animal Hyaluronic Acid Content

Table 1 delineates the findings on the concentrations of sugars/polysaccharides, HA, uronic acid, N-acetylglucosamine (NAG), and water present within the non-animal hyaluronic acid derived from *Tremella fuciformis*. What is evident from the data (Table 1) is that the extract obtained from *Tremella fuciformis* is significantly enriched in polysaccharides, as the concentration of these compounds within the extract is remarkably high, constituting approximately 90.6% of the total weight of the extract. This observation implies that, once the extraction protocol is refined, it may be a pivotal method for obtaining polysaccharide extracts with exceptionally high yields. Upon establishing that the extract resulting from the extraction procedure was indeed a polysaccharide extract from *Tremella fuciformis*, it became imperative to elucidate the characteristics of the polysaccharides. The analysis revealed that of the 90.6% polysaccharides present in the extract, approximately 87.8% were identified as HA, indicating that the polysaccharide extract acquired from *Tremella fuciformis* is notably abundant in HA. Subsequently, the uronic acid content was less than 3% residual, signifying an advantageous yield from the extraction process. The scientific literature corroborates this finding, since Li and colleagues conducted a study to evaluate the effect of different drying methods on the chemical composition and physical properties of *Tremella fuciformis* extracts. Specifically, it was observed that a low-temperature drying process (freeze-drying) could maintain the uronic acid content (2.24%) at levels comparable to that of the undried mushroom (2.87%) [43]. Similarly, the NAG content was also evaluated. The reported data demonstrate a minimal presence of NAG within the extract, which was found to be less than 0.1%. Finally, the water content within the extract was determined, which was observed to be about 7.6% of the total extract.

In conclusion, the results obtained from the analysis of the non-animal hyaluronic acid from *Tremella fuciformis* showed a high concentration of polysaccharides, particularly HA, which constituted about 87.8% of the total polysaccharides in the extract. These data suggest that the extraction process permits powder extraction of hyaluronic acid or similar molecules. Furthermore, the low presence of NAG residues and the relatively low water content indicate a good efficiency of the extraction process, making this extract a promising candidate for industrial applications.

#### 3.1.2. Analysis of Metals and Sodium Content

Metals including arsenic, cadmium, cobalt, chromium, copper, lead, mercury, zinc, nickel, tin, selenium, iron, magnesium, and manganese may be present within the sediments of mushroom cultures, consequently influencing their overall composition. Hence, rigorous screening for heavy metals was conducted to eliminate the possibility of contamination in the non-animal hyaluronic acid extract from *Tremella fuciformis*. In Table 2, all analytical data pertinent to the metals are presented, revealing either the absence of heavy metals (arsenic, cadmium, cobalt, chromium, mercury, lead, copper, and zinc) or their presence in negligible quantities, alongside a minimal occurrence of non-heavy metals (nickel, iron, manganese, selenium, and tin), with the singular exception of magnesium, which was identified at a concentration of 99.8 ppm. Furthermore, the sodium content was analysed to ascertain that the extracted substance is not a salt derivative. The findings indicated that sodium levels comprised 1.77% of the extract in solution, thereby corroborating that the extract procured is indeed HA and not a derivative salt (e.g., Sodium Hyaluronate).

#### 3.1.3. Molecular Weight Determination

**Figure 2:**
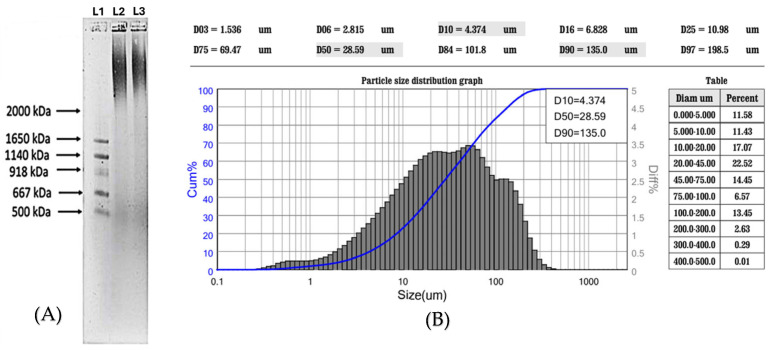
Analysis of molecular weight of non-animal hyaluronic acid. In (**A**), an example of 1% agarose gel. The sample loads are described by the following abbreviations: L1 = standard molecular weight HiLadder specific for HA detection; L2 = lane loaded with 3 mg/mL reference published [30]; L3 = lane loaded with 3 mg/mL non-animal hyaluronic acid extract. In (**B**), the chromatogram is obtained from the particle size distribution.

As illustrated in Figure 2A, the extract exhibited a molecular weight of over 2000 kDa, indicating that it resembles a high molecular weight variant of HA. As depicted in the sample chromatogram (Figure 2B), the results across all assessments revealed that the particle size distribution was recorded at Mn 5185 kDa and PD 2.364, supporting the data observed by agarose gel.

#### 3.1.4. Purity and pH Analyses of Non-Animal Hyaluronic Acid

**Figure 3:**
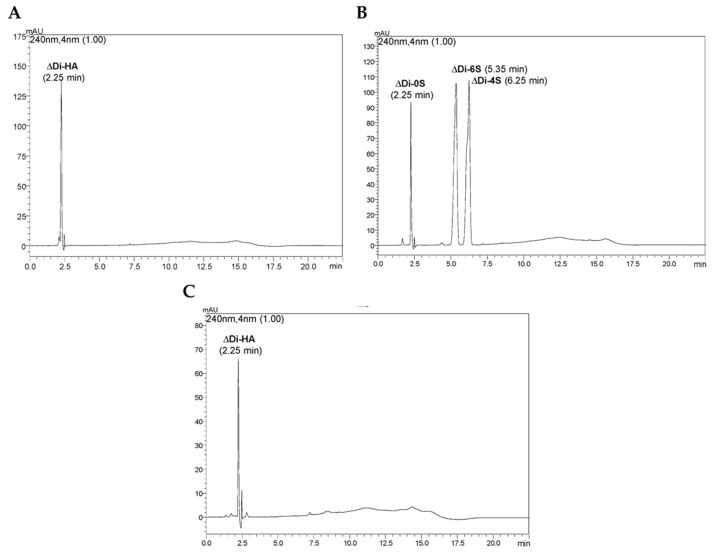
HPLC-UV and HRMS analysis of non-animal hyaluronic acid after enzymatic hydrolysis with chondroitinase AC. In (**A**), HPLC-UV chromatogram of non-animal hyaluronic acid sample and its positive HRMS spectrum. In (**B**), HPLC-UV chromatograms of a mixture of chondroitin disaccharides standard ΔDi-0S, ΔDi-4S, and ΔDi-6S, and in (**C**), a solution of the disaccharide standard ΔDi-HA of HA.

To understand whether non-animal hyaluronic acid was classifiable as fully fledged HA, purity was assessed by HPLC analysis to understand whether there were also sulfated polysaccharide forms. The analysis (Figure 3) revealed that the non-animal hyaluronic acid corresponded to disaccharide ΔDi-HA produced by enzymatic hydrolysis of Tremella extract with chondroitinase AC. The identity of this disaccharide was verified by comparison with the reference standard ΔDi-HA and the detection of protonated and sodium-plated positive ions in its mass spectrum. The same analysis also demonstrated the absence of chondroitin monosulfates 4 and 6, eluted at 6.25 and 5.35 min, respectively.

pH is a pivotal parameter that can considerably influence a variety of functional properties of a molecule, including colour, taste, and texture. In this study, the pH of *Tremella fuciformis* extract was measured and evaluated upon its dissolution in an aqueous solution. Furthermore, to maintain structural integrity, the HA must possess a pH exceeding 4 and fall below 11 when dissolved in solution; otherwise, the HA may undergo degradation. Based on the analysis conducted by the USP 791 methodology (Table 3), the non-animal hyaluronic acid exhibits a pH value approximating 6.5 pH units, which resides within the acceptable range for application (5.5–7.5).

### 3.2. Physical Characterisation Results of Non-Animal Hyaluronic Acid

To verify that non-animal hyaluronic acid was indeed of quality following all extraction steps, the following experiments were performed to characterise the structure of this extract: NMR analysis, IR/ATR spectrum, and MALDI-TOF analysis [41,44].

#### 3.2.1. NMR Analysis of Non-Animal Hyaluronic Acid

**Figure 4:**
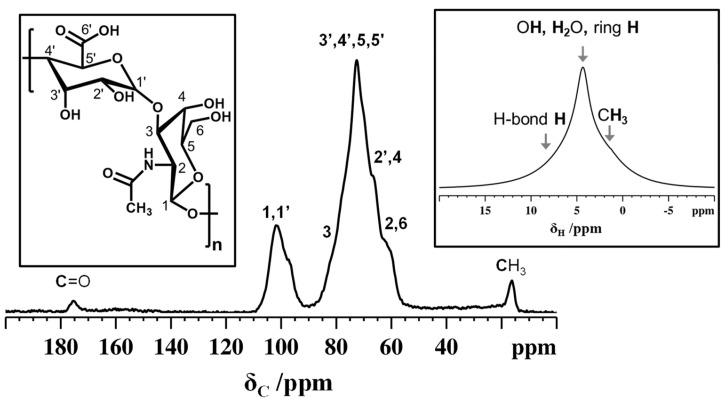
^13^C CPMAS NMR spectrum of non-animal hyaluronic acid recorded using a MAS rate of 11 kHz and a CP contact time of 0.5 ms. Inset (**right**) shows the ^1^H MAS NMR spectrum of non-animal hyaluronic acid recorded using an MAS rate of 15 kHz. Inset (**left**) shows the molecular structure of hyaluronic acid.

Solid-state NMR spectroscopy is a valuable tool for investigating the molecular structure, composition, and functional groups of polysaccharides in solid state. By studying ^13^C CPMAS NMR, it is possible to detect important structural elements and 3D arrangements of polysaccharide chains [45]. In the ^13^C CPMAS spectrum (Figure 4) of non-animal hyaluronic acid, ^13^C peaks appeared very broad due to carbohydrate polymer chains in a disordered amorphous state. The methyl carbon appeared at around 16.5 ppm while the carbonyl carbon resonance was around 175.5 ppm. Moreover, the very broad peak observed between δ = 55–85 ppm is linked to the different ^13^C sites in the disaccharides, which consist of glucuronic acid and N-acetyl-glucosamine monomeric units. The disaccharide’s C1 and C1’ peaks appeared at around 101 ppm. The polymer chains of the disaccharides are linked via the glycosidic bonds forming interconnected networks constituting several randomly ordered three-dimensional arrangements. The broad downfield peak detected in the ^1^H MAS NMR spectrum (Figure 4) confirmed the extended intra- and interchain hydrogen bonds responsible for the disordered arrangements.

The ^13^C chemical shifts and number of peaks for non-animal hyaluronic acid are similar to those reported in the literature. The only difference is related to the ^13^C NMR peak resolution. However, the ^13^C NMR spectra reported in the literature were for animal-derived sodium hyaluronate, while here we report the hydrated form of non-animal hyaluronic acid [46,47].

#### 3.2.2. ATR-FTIR Spectroscopy

**Figure 5:**
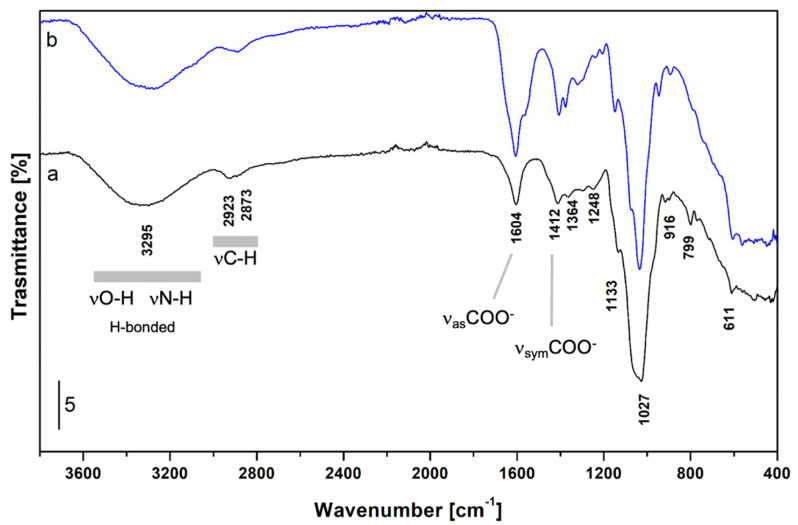
ATR-FTIR spectrum of non-animal hyaluronic acid (curve a, black) and standard hyaluronic acid (curve b, blue).

The ATR-FTIR spectrum of the studied extract was measured to validate the structural and compositional data of non-animal hyaluronic acid. Attenuated Total Reflectance (ATR) is a widely used sampling method in FTIR spectroscopy, employing total internal reflection to generate an evanescent wave that penetrates the sample surface. This wave allows detailed data acquisition on molecular composition and structure. ATR’s popularity stems from its versatility in analysing both solid and liquid samples in their native state, streamlining the analysis of a wide range of substances. The results showed that the spectrum (Figure 5, curve a) is fully compatible with the structure of hyaluronic acid in hydrated form, with the most significant bands assigned as follows. The broad band in the 3600–3000 cm^−1^ range (peak at 3295 cm^−1^) is due to hydrogen-bonded O-H and N-H stretching modes; signals at 2923 and 2873 cm^−1^ are assigned to asymmetrical and symmetrical stretching modes of C-H groups in CH, CH_2_, and CH_3_ groups. In the low frequencies’ region, bands at 1604 and 1412 cm^−1^ are ascribed to the ν_as_ and ν_sym_ of COO^−^ groups, respectively; the shoulder at 1645 cm^−1^ is due to the bending mode of water and the amide I band. The signals at 1133, 1048, and 1027 cm^−1^ are assigned to C-O-C in glycosidic groups, C-O (exocyclic), and C-OH groups, respectively. For the sake of comparison, the ATR-FTIR spectrum of standard HA is reported (Figure 5, curve b). In summary, it was observed that apart from some differences in relative intensities (related to possible differences in chain length and particle size of the sample), the same peaks identified in the non-animal hyaluronic acid may be traced back to what is known in the literature for standard HA [48,49,50].

#### 3.2.3. MALDI-TOF

**Figure 6:**
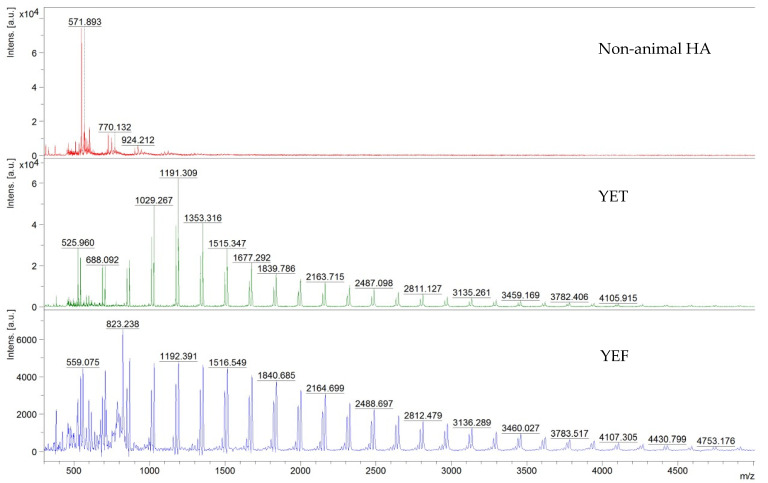
Representative MALDI-TOF mass spectra in the 300–5000 *m/z* region. The abbreviations include the following: YET = extract of non-animal hyaluronic acid before extraction; YEF = intermediate extract of non-animal hyaluronic acid; non-animal HA = non-animal hyaluronic acid.

MALDI-TOF mass spectrometry was used to analyse different steps during non-animal hyaluronic extract preparation. The characterisation of HA with MALDI mass spectrometry presents some challenges due to its chemical properties. HA has poor ionisation efficiency, and optimising sample preparation can improve characterisation. However, direct analysis of highly polydisperse mixture of long HA fragments (>10 kDa) is complicated due to short fragments suppressing high mass species ions. In our analysis, the final extract was compared with an extract of non-animal hyaluronic acid before extraction (designated as YEF) and with an intermediate extract (designated as YET). The findings illustrated in Figure 6 and Table A3 (reported in Appendix B) demonstrate variances in the peaks population. Elevated peaks characterise the initial pulverised YEF in comparison to the intermediate YET. In both samples (YEF and YET), some peaks corresponding to HA oligomers were detectable, in detail 1516.55 and 1515.37 corresponding to 8 mer; 2650.58 and 2649.02 corresponding to 14 mer. The molecular weight difference between the high-intensity peaks is quantified at 162 Da, corresponding to a single glucan unit. The minimal difference is quantified at 17 Da, attributed to chlorine adducts or hydroxyl groups. Another way to interpret the MALDI-TOF spectra is by observing that glucans were detected as sodium ions adducts ([Glcn + Na]^+^) displaying a peak-to-peak mass differential of 162 Da, which is consistent with the repetitive unit of β-(1→3) glucan [32]. Similar findings have been documented previously [49]; the fruiting body of *Tremella fuciformis* is constituted of (1→2)- and (1→4)-mannose as well as (1→3)-linked glucans [21]. Based on the data obtained, it can be discerned that there is a progressive and pronounced decrease in the number of peaks from the raw material YEF to the extract of non-animal hyaluronic acid. Indeed, a molecular weight disparity is observable between the high-intensity peaks corresponding to a sugar unit, specifically glucan. This substantiates the efficacy of the extraction and purification methodology employed to obtain the non-animal hyaluronic acid, which is more prominently present in the sample preceding digestion (YEF sample) through an enzymatic process, as represented by an intermediate extract (YET sample). Ultimately, the purified non-animal hyaluronic acid significantly diminishes the number of units discernible as glucan.

## 4. Discussion

*Tremella fuciformis*, commonly called snow fungus or silver ear fungus, is an edible medicinal mushroom from the *Tremellales* order and the *Tremellaceae* family. It contains various bioactive compounds, including fatty acids, proteins, enzymes, polysaccharides, phenols, flavonoids, fiber, and trace elements [4]. The polysaccharides in the fruiting bodies, spores, mycelia, and fermentation liquors are particularly important. Their composition and molecular weight influence their properties, which vary from 8 × 10^3^ to 6 × 10^6^ Da. The composition depends on the extraction method, with sugars such as fucose, galactose, xylose, mannose, glucose, and glucuronic acid involved [51]. Different extraction techniques are used depending on the desired application of the extract [4,52]. For instance, hot water extraction of the fruiting body results in mannose with (1→2)- and (1→4)-linked structures, as well as (1→3)-linked glucans. The mycelium, in contrast, contains mannose, rhamnose, glucuronic acid, and glucose in various ratios.

Additionally, polysaccharides from *Tremella fuciformis* include xylose, mannose, and glucuronic acid linked by α-1,3-glycosidic bonds, with side chains of galactose, arabinose, and trace amounts of fucose [21]. The extract also has a significant amount of uronic acid (mainly glucuronic acid) and hydroxyl groups, which help maintain its stability over time, preserving its physical and chemical properties like electrical conductivity and pH [51]. Polysaccharides are classified into five types: acidic polysaccharides, neutral polysaccharides, acidic oligosaccharides, and cell wall polysaccharides [53]. Studies suggest that the viscosity of these polysaccharides plays a critical role in their biological activity, affecting their function in the body [54,55].

Based on this evidence, the following study set itself the goal of chemically and physically characterising a polysaccharide extract obtained from *Tremella fuciformis* to assess whether it was a type of extract that could be traced back to hyaluronic acid. Extraction of HA from solid tissues involves several steps: digestion (protease), boiling (denatured enzyme), centrifugation, extraction with chloroform, centrifugation, dialysis, precipitation with ethanol, centrifugation, redissolving, digestion, and boiling for denaturation of the enzyme. In addition, HA extraction techniques use detergents, enzymes, and/or solvents to break down the structure and isolate polysaccharide complexes from the tissue. These techniques depend on the chemical hydrolysis of the tissue, resulting in the breakdown of the proteoglycan core [16]. The extraction method proposed in this study does not incorporate a centrifugation step. Instead, an ethanol removal and pasteurisation step was performed after the ethanol and water precipitation step. This approach yielded a polysaccharide extract that exhibited comparable properties to hyaluronic acid, a result that is not attainable through conventional extraction methodologies. Notably, *Tremella fuciformis* extract has been utilised as a natural substitute for hyaluronic acid. However, these properties were observed based on its physicochemical characteristics rather than its HA content, demonstrating superior effects compared to hyaluronic acid [20]. In the present study, the extraction process resulted in an almost totally pure HA extract from *Tremella fuciformis*, as will be discussed later. Indeed, on the chemical composition of the extract under scrutiny, the analysis revealed that the non-animal hyaluronic acid extract derived from *Tremella fuciformis* exhibited a marginally acidic pH (pH = 6.5), a property that is presumably attributable to the presence of acidic polysaccharides within the extract. This finding is analogous to the reported stability values of HA in aqueous solution. In the context of HA stability in aqueous solution at pH 7, it has been observed that the degradation rate increases exponentially with rising temperature. Conversely, an acidic pH environment has been shown to cleave the HA molecule, leading to the release of NAG [56]. In the present study, the stability of a non-animal HA molecule in solution at pH 6.5 was investigated by determining the presence of NAG. The data obtained demonstrated a reduced presence of NAG, significantly affecting the stability of the high molecular weight polymer.

Furthermore, a high amount of polysaccharides was observed in the non-animal hyaluronic acid (about 90.6%), showing a similar yield value to those reported in the literature (about 89.6%) [35]. Given the hypothesis that the subject extract is abundant in hyaluronic acid, this substance’s content was determined by a colorimetric technique. This analysis method agreed with the data obtained by HPLC analysis. Indeed, non-animal hyaluronic acid demonstrated an HA content of approximately 87.76%, a profile comparable to the published reference data (about 95%) [30], as well as the HPLC analysis that revealed the presence of HA, which was detected as the corresponding disaccharide ∆Di-HA generated by chondroitinase AC enzymatic hydrolysis of the *Tremella fuciformis* extract. The characterisation of this disaccharide was determined through comparative analysis with the reference standard ∆Di-HA, alongside the identification of protonated and sodium cations observed in its mass spectrometric profile.

These data confirm the presence of hyaluronic acid in the extract, suggesting the success of the extraction process. Furthermore, the analysis of NAG also showed the success of the extraction process, as it was almost non-existent (value of less than 1%). Finally, it is important to mention that the characterisation of the non-animal hyaluronic acid content confirmed the presence of hyaluronic acid by assessing the molecular weight distribution and particle size. This finding corroborates the data reported in the extant literature (higher molecular weight over 2000 kDa) in which the polysaccharides are likened to hyaluronic acid for its application in the food, pharmaceutical, and cosmetic industries [30,57].

In addition, the data obtained by analysing the metal screening, sodium, and water content supported previous data on the presence of hyaluronic acid, excluding its salt (sodium hyaluronate); indeed, water content was about 7.6%, while sodium content was less than 2% (about 1.77%). Regarding the metal levels, the metals proved to be below the limit for human use, confirming the extraction process’s safety.

Further tests were performed to confirm the composition of non-animal hyaluronic acid. Related to NMR analysis, the natural abundance of ^13^C in non-animal hyaluronic acid revealed that the spectrum was compatible with HA structure, as reported in the literature [41,46,47]. In addition, the ATR-FTIR spectrum showed some differences in relative intensities (correlated with possible differences in chain length and sample granulometry). Still, the peaks identified in non-animal hyaluronic acid were similar to what was observed in the literature, thus confirming its identity [44].

Furthermore, non-animal hyaluronic acid has a molecular weight of over 2000 kDa; in this case, the direct analysis by MALDI mass spectrometry is complicated by the presence of short fragments ionisation. The MALDI-TOF analysis incorporated *Tremella fuciformis* powder, the extract after the first step of the extraction process, and the final non-animal hyaluronic acid to assess disparities in polysaccharides. A detailed observation of the MALDI-TOF spectra revealed discrepancies in the peak population. The YEF (YEF powder) sample had more peaks than the YET (initial stage of the extraction process). The visible difference in molecular weight between high-intensity peaks was 162 Da, corresponding to one glucan unit and consistent with the b-(1 3)-glucan repetition unit [49,58]. Based on the data obtained, it can be stated that there has been a progressive and marked reduction in the number of peaks from raw material (YEF powder) to non-animal hyaluronic acid, which is observable in molecular weight between the high-intensity peaks corresponding to one unit of sugar, in particular glucan. This demonstrates the correct extraction and purification procedure to obtain non-animal hyaluronic acid.

In conclusion, this comprehensive chemical and physical characterisation of *Tremella fuciformis* significantly enhances our prior investigations regarding its advantages for joint and dermal health. By systematically analysing its chemical and physical attributes, we aim to substantiate further the burgeoning evidence concerning Tremella’s efficacy as a supplement to enhance overall well-being. This research advances our comprehension of its potential, bridging the divide between its biological effects and practical applications in health maintenance.

## 5. Conclusions

In conclusion, the non-animal hyaluronic acid extract from *Tremella fuciformis* (GreenIuronic^®^) under consideration in this study has proven to be a promising source of hyaluronic acid or structurally similar polysaccharides with significant potential in the cosmetic and pharmaceutical industries. Preliminary chemical and physical analyses have confirmed the presence of bioactive components, including hyaluronic acid, within the extract. Even though preliminary stability analyses of the extract have been conducted and documented in Appendix B (see Table A4), further research is required to substantiate this stability and determine its efficacy over extended periods of utilisation. Furthermore, molecular characteristics, including the molecular weight and composition of sugar, have been determined to be crucial for the functional properties of the extract, suggesting that careful selection of the extraction method can optimise its benefits. Notwithstanding the positive results demonstrated in this study, several significant issues must be considered in future research aimed at improving the characterisation of *Tremella fuciformis* extract and verifying further applications in the cosmetic and pharmaceutical sectors. The characterisation of the extract will be further investigated, including possible variations in the chemical composition and functional properties of the extract in response to different cultivation and extraction parameters.

## Figures and Tables

**Table 1:** Data related to total polysaccharides, HA, uronic, NAG, and water content are expressed as % *w*/*w* ± SD. For all analyses, three independent experiments were carried out in quadruplicate. I.M. = internal method; F.K.W.C = Karl Fisher water content.

Analysis	Value	Unit of Measure
Total polysaccharides (I.M.)	90.6	% (*w*/*w*)
Total Hyaluronic Acid (I.M.)	87.8	% (*w*/*w*)
Uronic content (I.M.)	2.39	% (*w*/*w*)
NAG content (I.M.)	<0.1	% (*w*/*w*)
Water Content (F.K.W.C.)	7.6	% (*w*/*w*)

**Table 2:** Metals analysis performed on USP 233. For all analyses, three independent experiments were carried out in quadruplicate. The indicated measurement uncertainty corresponds to the expanded uncertainty with coverage factor k = 2 at a probability level *p* = 95%. The laboratory does not correct the analytical results for the recovery factor. The decision rules adopted for the expression of conformity (if any) do not consider the associated uncertainty contribution unless specifically requested by the customer to take it into account or specifically required by the relevant legislation or regulation. When preceded by the symbol “<”, the result refers to the lower limit of quantification of the applied method.

Analysis	Value	Unit of Measure
Arsenic (USP <233>)	<0.5	ppm
Cadmium (USP <233>)	<0.5	ppm
Cobalt (USP <233>)	<0.5	ppm
Chromium (USP <233>)	0.88	ppm
Iron (USP <233>)	9.76	ppm
Magnesium (USP <233>)	99.8	ppm
Manganese (USP <233>)	1.16	ppm
Mercury (USP <233>)	<0.2	ppm
Nichel (USP <233>)	<0.5	ppm
Lead (USP <233>)	<0.5	ppm
Copper (USP <233>)	<0.5	ppm
Selenium (USP <233>)	<1	ppm
Tin (USP <233>)	<0.5	ppm
Zinc (USP <233>)	<0.5	ppm
Sodium (USP <233>)	1.77	% (*w*/*w*)

**Table 3:** pH analysis. The value is a pH unit of three independent experiments carried out in quadruplicate.

Analysis	Value	Unit of Measure
pH (USP <791>)	6.5	Unit of pH

## Data Availability

The Laboratory of Physiology (F.Uberti) collects raw data and takes appropriate procedures to preserve them in a secure system forever. The corresponding author can provide this study’s data upon reasonable request.

## Abbreviations

The following abbreviations are used in this manuscript:

TFPS	polysaccharide of *Tremella fuciformis*
HPLC	high-performance liquid chromatography
HPLC-UV	high-performance liquid chromatography-ultraviolet
HPLC-HRMS	high-resolution mass spectrometry
UV	ultraviolet spectroscopy
IR	infrared spectroscopy
ATR	attenuated total reflectance
GC	gas chromatography
MS	mass spectrometry
NMR	nuclear magnetic resonance
GalNAc	acetylgalactosamine
NAG	N-acetylglucosamine
USP	United States Pharmacopeia
HA	hyaluronic acid
D_2_O	deuterium oxide
FTIR	Fourier transform infrared spectroscopy
MALDI-TOF	matrix-assisted laser desorption/ionisation time-of-flight

## Appendix A

**Table A1:** Gradient elution program.

Time (min)	Mobile Phase B%
0.00	20
7.00	65
12.00	65
12.50	20
22.50	20

**Table A2:** Gradient elution program implemented.

Time (min)	Mobile Phase B%
0.00	20
4.00	65
9.50	65
10.00	20
15	20

## Appendix B

**Table A3:** Peaks list of representative MALDI-TOF spectra in the 300–5000 *m/z* region. The abbreviations include the following: YET = extract of non-animal hyaluronic acid before extraction; YEF = intermediate extract of non-animal hyaluronic acid; non-animal HA = non-animal hyaluronic acid.

Non-Animal HA	YET	YEF
*m/z*	*m/z*	*m/z*
312.697	381.001	381.103
330.624	454.668	459.885
374.738	458.837	464.996
462.842	464.888	478.402
510.252	478.390	524.831
532.431	482.196	543.104
543.860	495.822	559.075
565.865	502.348	581.223
571.893	517.972	598.114
580.831	525.960	614.146
587.856	543.142	636.220
593.898	565.258	675.538
598.787	582.102	687.308
603.820	613.764	705.572
609.718	620.098	713.488
719.971	668.689	751.652
770.132	688.092	765.576
902.166	705.114	779.085
924.212	778.659	785.838
945.997	833.094	796.461
1122.485	850.682	806.889
	867.190	823.238
	1029.267	849.652
	1175.314	867.906
	1191.309	995.427
	1337.360	1011.832
	1353.316	1030.180
	1499.363	1157.125
	1515.347	1174.062
	1661.297	1192.391
	1677.292	1319.139
	1823.270	1336.227
	1839.786	1354.493
	1984.827	1481.139
	2001.761	1498.332
	2146.814	1516.549
	2163.715	1643.134
	2309.045	1660.513
	2325.651	1678.650
	2471.000	1805.220
	2487.098	1822.475
	2633.236	1840.685
	2649.024	1967.455
	2794.832	1984.535
	2811.127	2002.688
	2956.619	2129.519
	2972.900	2146.446
	3118.915	2164.699
	3135.261	2291.634
	3280.830	2308.450
	3296.715	2326.695
	3442.620	2452.915
	3459.169	2470.398
	3604.503	2488.697
	3620.750	2615.662
	3766.399	2632.355
	3782.406	2650.580
	3928.011	2777.754
	3944.584	2794.233
	4090.139	2812.479
	4105.915	2921.118
	4267.371	2939.020
		2956.096
		2974.430
		3101.218
		3118.087
		3136.289
		3262.422
		3280.065
		3298.096
		3424.925
		3441.606
		3460.027
		3586.663
		3603.239
		3622.009
		3765.512
		3783.517
		3926.940
		3945.246
		4088.691
		4107.305
		4251.836
		4269.047
		4412.032
		4430.799
		4592.707
		4735.711
		4753.176
		4915.806

**Table A4:** Data related to HA content are expressed as % *w*/*w* ± SD (analyses performed every two months for one year).

Non-Animal HA	May 2023	August 2023	November 2023	February 2024	May 2024
HA content	92.0%	91.2%	91.2%	88.2%	87.8%
